# Visual communication design: a neglected factor in nutrition promotion via packaged food labels

**DOI:** 10.3389/fpubh.2024.1296704

**Published:** 2024-02-14

**Authors:** Meghan Kelly, Jennifer R. McCann, Celeste I. Chapple, Julie Woods, Catherine G. Russell

**Affiliations:** ^1^School of Communication and Creative Arts, Faculty of Arts and Education, Deakin University, Geelong, VIC, Australia; ^2^School of Exercise and Nutrition Sciences, Institute for Physical Activity and Nutrition (IPAN), Deakin University, Geelong, VIC, Australia

**Keywords:** public health, nutrition policy, food packaging, communication, design, food labeling, claims, consumer behavior

## Abstract

Packaging design is a communication device and a critical component in branding strategy, and has relevance for food policy. Presently, packaging-related nutrition policy initiatives focus on the role of regulated claims, nutrition information panels and front-of-pack nutrition labels to help guide consumer food choices and address high prevalences of discretionary and ultra-processed food consumption in many countries. However, these nutrition labelling systems are not optimized as public health policy tools as many consumers do not use them to inform their food choices. Visual communication design theory posits that a designer orders the elements and principles of design into hierarchies that prioritize certain elements over others, and that some of these elements are more dominant and given more emphasis than others. The overall design of the package thereby directs consumer attention to some aspects of pack design (e.g., characters, contents of the package) and away from others (e.g., nutrition details). Dual processing frameworks propose that food decisions are made with the interplay between automatic and rational thinking processes. Packaging designs affect whether consumers rely predominantly on automatic or rational thinking to select a food. This narrative review outlines the role of food packaging design and how it impacts the clear communication of nutrition aspects of food products and how the use of nutrition information by consumers to make decisions may depend upon design structures in packaging. This article attests that nutrition scientists and policy makers should incorporate visual communication design into research on the food packaging as a public health promotion tool. A stronger focus on the communication of regulated front-of-pack nutrition information can be made with a re-evaluation of the hierarchy of elements in the front-of-pack design enabling consumers to make healthier decisions.

## Introduction

Discretionary and ultra-processed foods form a large proportion of contemporary diets (1–5). These foods are often consumed as packaged foods, typically purchased relatively cheaply from grocery stores/supermarkets (6, 7), thereby contributing to the high prevalence of low-quality diets and attendant elevated non-communicable disease risk (8, 9). Food packaging designs motivate purchase and consumption, and are therefore one way by which food environments influence food choice and consumption and may be an important reason why consumers make unhealthy food choices (10). Considering the overall pack design, including both the use of mandatory or voluntary nutrition labels along with other design elements, and how these influence consumer decision making processes, could provide new avenues for optimizing the use of nutrition labels by consumers.

For nutrition labelling to be effective it is pertinent that the complete nutrition and health properties of the product are clearly available on food packages so consumers notice and use this information to make informed decisions. Dual-processing frameworks provide a useful way to understand consumer responses to food labelling and packaging designs (11–16). According to these frameworks two interacting processes are involved in decision making: System 1 (or bottom-up) and System 2 (or top-down) processes. System 1 processes are fast, intuitive, emotive and informed by learned associations and emotions. In contrast, System 2 processes are slower, reflective, controlled, and conscious, typically linked to goal-directed behaviors (12, 17). According to these frameworks, packaging attributes interact with consumer characteristics and contexts to determine whether the packaging is effective at promoting healthier food choices. The mechanism by which this occurs is whether System 1 or System 2 processing is more dominant during the choice process. For food packaging, the use of emotive claims, mascots/celebrities, attractiveness of the overall visual design, colors and images of products or other visual cues can evoke System 1 processing. Conversely, nutrition information panels and nutrition and health claims would be expected to require effortful System 2 processing.

Contextual factors, level of involvement in making the decision, goals, and individual characteristics play important roles in determining whether System 1 or System 2 is more dominant in decision making. In circumstances where level of involvement is lower due to reduced cognitive resources (e.g., time pressure, consumers are fatigued), or consumers are not motivated to select a healthier option (e.g., instead seeking a food reward), they may be less likely to rely upon System 2 (and therefore on-pack nutrition information) (15). In current regulatory contexts where consumers must disentangle regulated nutrition information from the other elements of packaging design (including voluntary nutrition claims), and where marketing cues can be incongruent with the product’s nutrition profile (18), it is expected that consumers need to engage System 2 processing to use on-pack nutrition information and make healthy food choices (14). However, for on-pack nutrition information to be a more effective health promotion policy tool, packs need to be designed in ways that recognize dual processing perspectives of consumer decision making. Packs should be designed in ways that either promote System 2 processing by directing consumer attention to regulated nutrition information, or System 1 processing whereby marketing and other design elements are congruent with the product’s nutrition profile and therefore can inform choices. Understanding of the various elements on food packages, including nutrition labels and marketing elements, as well as visual communication design principles that encompasses what, where and how these elements are arranged, can provide new avenues for promoting healthy diets through on pack nutrition labeling.

There is a long-standing history in the use of both mandatory and voluntary nutrition labelling practices to promote healthier diets, with the World Health Organization (19) and United Nations (20) and a range of other more localized bodies including Food Standards Australia New Zealand (21, 22) and the European Union (23) recognizing the important role of food labelling in affecting food environments and therefore diets. Historically, nutrition information/facts panels, which typically provide information on average energy, total fats, saturated fats, carbohydrates, protein and sodium per 100 grams and/or serve of product, were the main method to provide nutrition information on products. However, this information requires knowledge, time, and effort to use, and is typically not on the front of a package, meaning it requires effort to locate and use. This led to the introduction of simpler, more interpretive, front-of-pack labels (e.g., Health Star Rating (HSR) in Australasia, Nutri-Score in parts of Europe or warning statements in South America) designed to summarize nutrition information about the products and make it readily visible and understandable to a wider range of consumers. Product manufacturers also use voluntary nutrient and health claims on the front of packages to communicate nutrition and health information. In this regard, product manufacturers promote products through the exclusive use of positive statements (claims) and do not make transparent potentially problematic nutrients such as high levels of added sugar or salt (24–27).

A large body of work has investigated how these various nutrition or health labels influence consumer perceptions, awareness, understanding, and use. This primarily includes front-of-pack labels, nutrition, health and related claims, and nutrition information panels (28), including, more recently, warning labels (29–31) and “high in” labeling (32). This work has been extensively reviewed elsewhere [see (30, 33–39)]. Empirical findings on the efficacy of these elements to influence perceptions and choice are mixed (29–31, 34, 35, 37–43): it appears that the effects of nutrition information on food packages may depend upon a range of factors including the type of information presented on the pack, the way in which the information is presented (e.g., numerically, visually, using particular colors), and how these interact with the characteristics of the consumer, the context in which the food decisions are being made the type and what other information is present Therefore, although nutrition labels have promise as a policy tool to influence consumer behaviors, they are not yet optimized for use by all consumers across a range of foods and contexts.

There is also a collection of somewhat disparate other research studies related to understanding how packaging designs influence consumer food choices. This body of work has considered the effects of simultaneous presentation of various claims as part of an over pack design, the combined/interacting effects of claims and nutrition information (44, 45), or the role of claims in biasing health perceptions (46). Further research has examined the impact of food package graphic design on consumers, with these studies showing the utility of graphic design for being a useful tool in communicating consumer benefits (47), that the positioning of packaging elements influences consumer attention (48), and that a relationship between packaging design and willingness to pay exists (49). Additionally, food pack design research has shown that consumers are able to process multiple packaging messages concurrently (47), that visual design cues are an important influence on consumer choices (10, 50, 51), and that brand and other visual cues such as colors and images can influence choices (52–55). There is also a recognition that other aspects of packaging design including auditory, haptic, and olfactory characteristics influence consumers (56) and that physical features (e.g., shape) influence attention in crowded marketplaces (56, 57). There are also studies (18, 58) examining implicit and explicit packaging design elements as heuristics, showing that these influence consumers differently. Taken together, these studies demonstrate the important role of design in influencing consumers in a range of ways. However, extant nutrition research on food packaging has neglected to sufficiently acknowledge the important role that visual communication design has on consumer-packaged food/drink choices and consumption behaviors. The aim of the present review is therefore to outline how design, including design hierarchy can be considered in food packaging research to promote healthy diets. This review does not aim to interrogate each of the elements or propose an alternative way to construct the arrangement of the visual elements of each package, nor will it seek to critique or alter the nutrition information already present on the package.

### Visual communication design’s role in consumer decision making

Packaging design, as a communication device, is a critical component in a branding strategy, one where the focus of a designer is to develop a positive relationship in a saturated retail environment, enticing consumers to make a purchase. As a specific area of design practice, food packaging design has become a communication device, offering information, and assuring consumers of their choice. The package becomes the living embodiment of a brand’s attributes, traits and personality establishing an inherent promise in the design of the package (59). “Packaging design is one of the key elements of a marketing strategy for a product as it is the visual face that will be promoted, recognized and sought out by the consumer” (60, p. 15). However, unlike manufacturers who use design to effectively market their products to consumers, the possibilities offered by design have not been exploited by nutrition scientists and policy makers. Therefore, it is vital to interrogate the many attributes present on food packages (e.g., claims, marketing images, brand names) and how packaging design is codified using design elements and principles to communicate to consumers. These are the tools that can be manipulated to promote healthier choices.

Visual communication design [i.e., the process of bringing a functional, esthetic, and organized structure to a group of diverse elements (61)] on food packaging can influence consumers at the point of purchase and impact whether on-pack nutrition information is used by consumers to make decisions (50, 62). Pack designs that capture attention and evoke emotions, generate product perceptions and expectations using color, fonts, imagery, and branding strategies can be used to direct consumers toward healthier alternatives (18). This could be via disrupting automatic (System 1) decision making to rely predominantly on rational (System 2) processes if designs direct attention to nutrition information. Alternatively, if nutrition information is designed and presented in ways that require little cognitive effort, then healthy decisions could be made by relying upon System 1 processes. The fundamental premise is that design is important to enhance the effectiveness of communication, increasing the capacity of the recipient to engage with the information and learn from the communication to make healthier choices.

#### Design elements and principles

Design elements and principles are the foundations of the language of design. Offering a definition of design elements and principles, Evans and Thomas (63) explain that the elements of design are the components that constitute the content of a graphic design composition while the principles of design are the way the components are placed together and the unseen forces that create interaction between the elements. Design elements are defined by Barnum et al. (64) as dot, line, shape, space, texture, value, size and scale, color, and typography whereas design principles are defined as balance, hierarchy, rhythm, pattern, unity, proportion, emphasis, and contrast. This list is not definitive, and a review of online resources and books will demonstrate different groupings and additional words defining design elements and principles; each are also correct and valid (65).

#### Visual design hierarchy

Design as a discipline uses signs, conventional and experiential, to communicate concepts to consumers (66). Visual communication designers work with codification of the visual to communicate and impart meaning in a very precise way. The consumer is an active participant in the exchange of information, entering a discourse (67). The purpose of design is to clearly communicate through visual information which involves choosing the right elements and crafting them in a way they communicate efficiently and effectively (68). Designers work with clearly defined design elements and principles that are organized into a system and placed within a context. It is by association to surrounding signs that meaning is created as the elements and principles work collectively to guide the viewer through the communication. Although the field of design continues to evolve with social change and rapid technological developments, and the growing need for market impact, the basic elements that are used to create the communication strategy remain the same (64).

Of relevance to nutrition information on food packages is the principle of hierarchy (the arranged order of elements) and the dominance (the relationship and influence of one element over another) or emphasis (the prioritizing of one element over another) in a design used to establish the path the viewer’s eye will take when they are presented with a food package (67). Once the consumer has looked at the dominant elements, and has become familiar with them, they seek the next level of communication and consider other elements that support the dominant elements (63). Designing with a clear emphasis on the dominant elements, followed by the secondary and subsequent support elements, reveals a meaning for the consumer. By managing the visual hierarchy, the designer controls how the design is read (63). The place of nutrition information in the visual hierarchy may impact whether it is used by consumers in System 1 and System 2 based decision making. That is, if nutrition information is placed low in the hierarchy, then it is unlikely that consumers will use this information when making decisions or forming impressions of products.

However, the overall design, its elements and their interrelations has not been examined as an influence upon attention to, and use of nutrition information in relation to food choices and intakes. We were unable to identify any papers that examined the way in which design structures, specifically design hierarchies, influence packaged food choices. This is problematic because for food packaging to be effective in promoting healthy diets, and ultimately good health, it needs to be able to influence the decision making of diverse consumers who purchase different foods in different contexts. We argue that design hierarchies are fundamental to doing this effectively by prioritizing clear and trustworthy nutrition information in pack designs.

#### Prioritizing clear and trustworthy nutrition information

It is apparent that by considering front of pack design hierarchy, novel ways of capturing and directing attention to nutrition information on food packages can be developed and tested as new avenues for improving public health. We propose this would generate new ways of designing front of packs that extend from focusing on what nutrition information is on the pack, toward a greater emphasis on where and how it is displayed, relative to other elements, and how this impacts consumers’ decision making as understood by dual processing models. Reducing the competition for attention (e.g., by reducing the prominence of marketing imagery) and strengthening those packaging elements that can promote health (such as nutrition information) is fundamental to improving health through packaged food choices. Designing food packages that effectively communicate nutrition information by using an understanding of the elements and principles of design can be generated and tested. In designing these packages, combining design principles with advances in understanding consumer decision making [e.g., dual processing theories (14), neural models (15)], to impact decision making could lead to novel approaches. Empirical research could test the effects of these new designs using existing methods (e.g., experiments, discrete choice studies, eye tracking) on packages both on consumer perception, attention, understanding, health inferences and choices, but also about packaging design appeal and brand strength as outcomes of interest to designers. Designs that enable consumers to make more accurate decisions with less cognitive effort (i.e., relying upon system 1) compared to current pack designs could be identified. As part of this, there is also an opportunity to address the need for the communication of both positive and negative nutrition information (e.g., via warning labels), in relation to other visual cues (e.g., marketing images), building on the comprehensive research on nutrition labels. The effects of these types of changes on different consumers and product categories needs to be tested.

**Figure 1:**
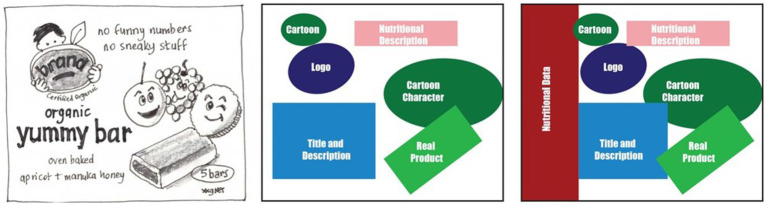
Illustrated image of child snack bar product (L) with elements highlighted with color (C) and proposed 25% of the pack dedicated to nutrition information (R).

**Figure 2:**
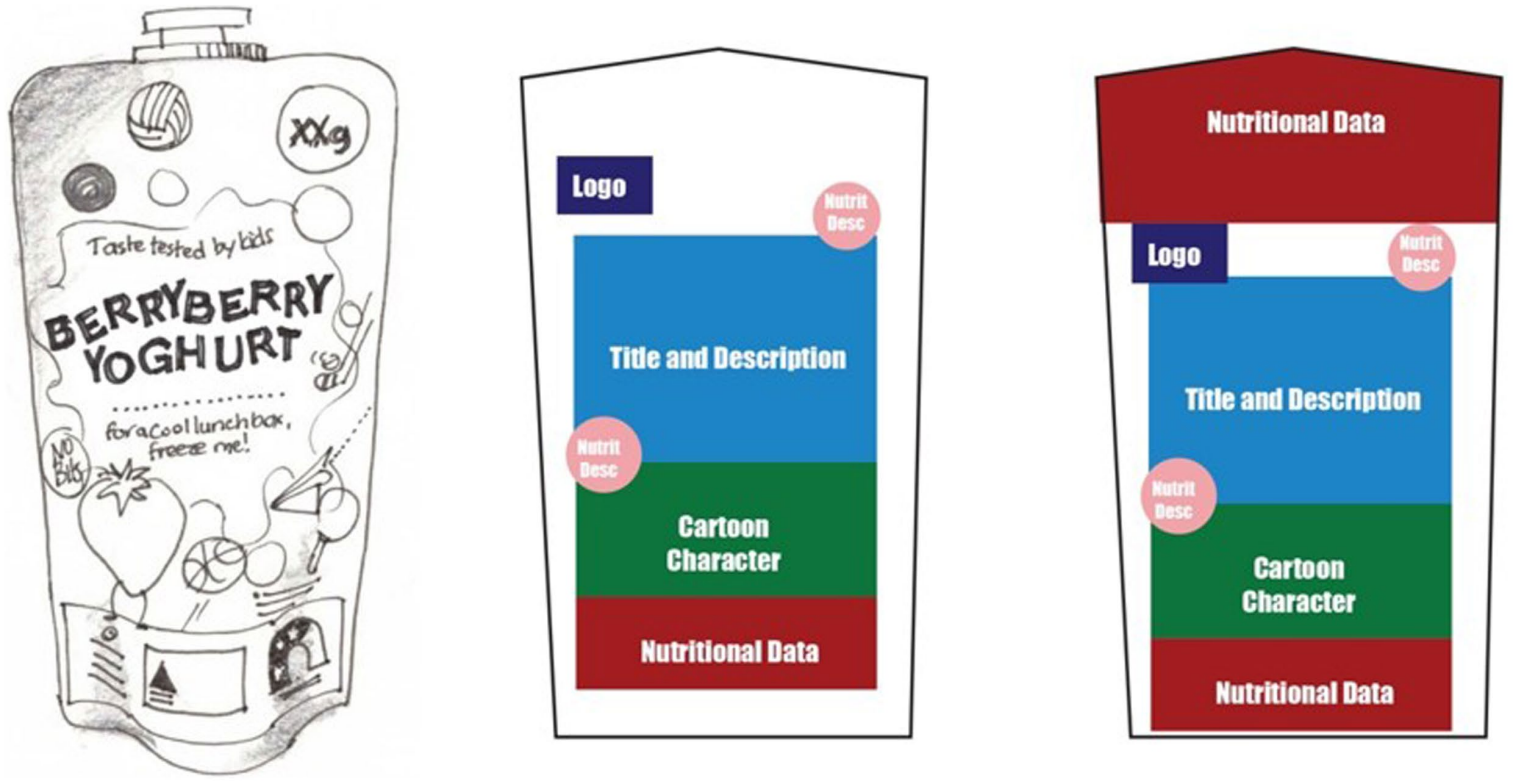
Illustrated image of pouch yoghurt product (L) with elements highlighted with color (C) and proposed 25% of the pack dedicated to nutrition information (R).

**Figure 3:**
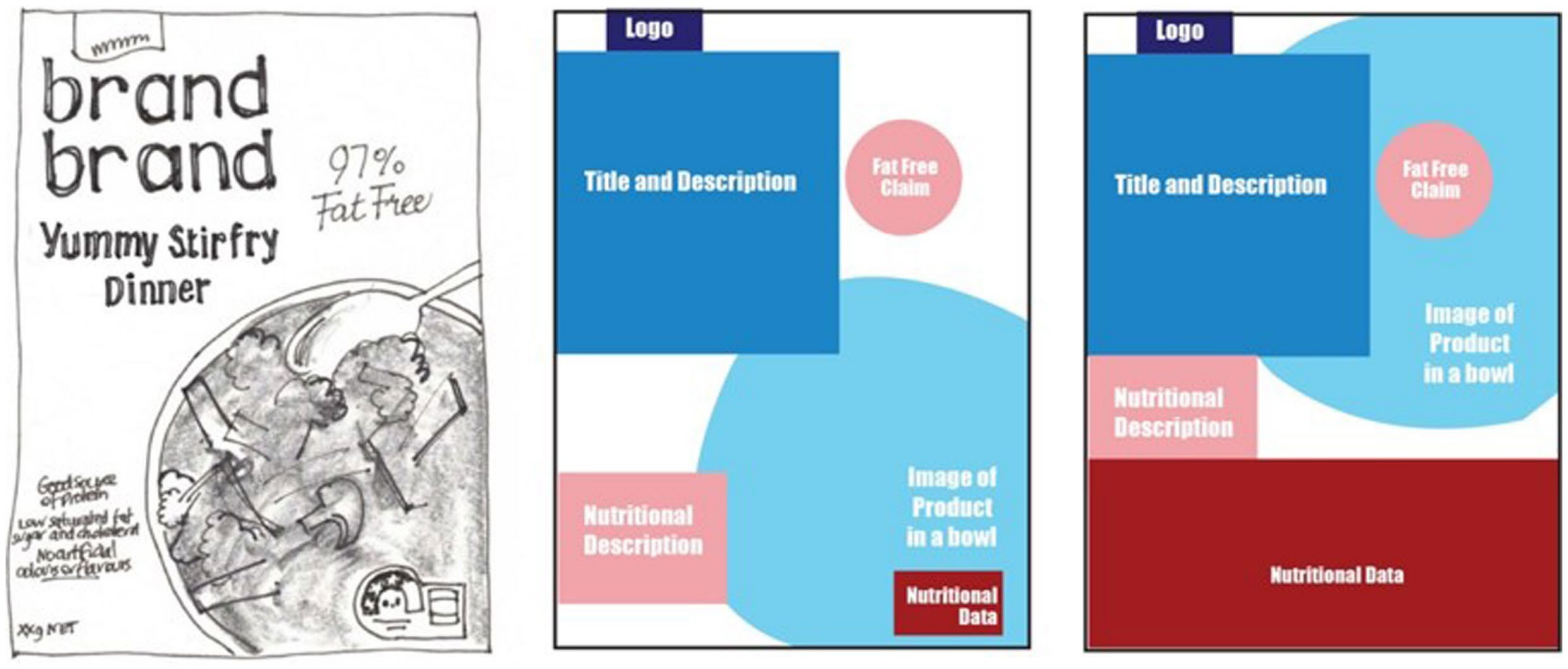
Illustrated image of readymade meal product (L) with elements highlighted with color (M) and proposed 25% of the pack dedicated to nutrition information (R).

Recognizing there are structural implications with the current approaches to packaging design, we propose that packaging designs should (i) increase the hierarchy of nutrition information and secondly, (ii) establish a consistent location for the information, and (iii) present objective information (both positive and negative). This could be addressed by removing most of the marketing information on packs (plain packaging approach). However, an alternative approach that allows for the preservation of branding and marketing information is to place a larger (e.g., 25% of packaging) panel of nutrition information on the front-of-pack design, moving existing design elements slightly to the left, right, up or down according to the package. Figure 1 below, demonstrates how this could be achieved using a typical snack bar found on Australian supermarket shelves. The nutrition panel has been located on the left of the package occupying approximately one quarter of the front panel. Each of the elements previously identified still appear on the package but have been pushed to the right. Figure 2 demonstrates how the nutrition information appears at the top of the pack, again taking one quarter of the front-of-pack space, moving the other elements lower in the packing design. Lastly, Figure 3 demonstrates one quarter of the front-of-pack hosting the nutritional information at the bottom of the package, moving the other elements higher. The principle of increasing the hierarchical impact and establishing a consistent location of the nutrition information is achievable in the examples below (Figures 1–3). For consumers, the benefits of considering design in this way is the increased capacity to identify healthier products with limited cognitive effort (i.e., by relying upon System 1).

**Figure 4:**
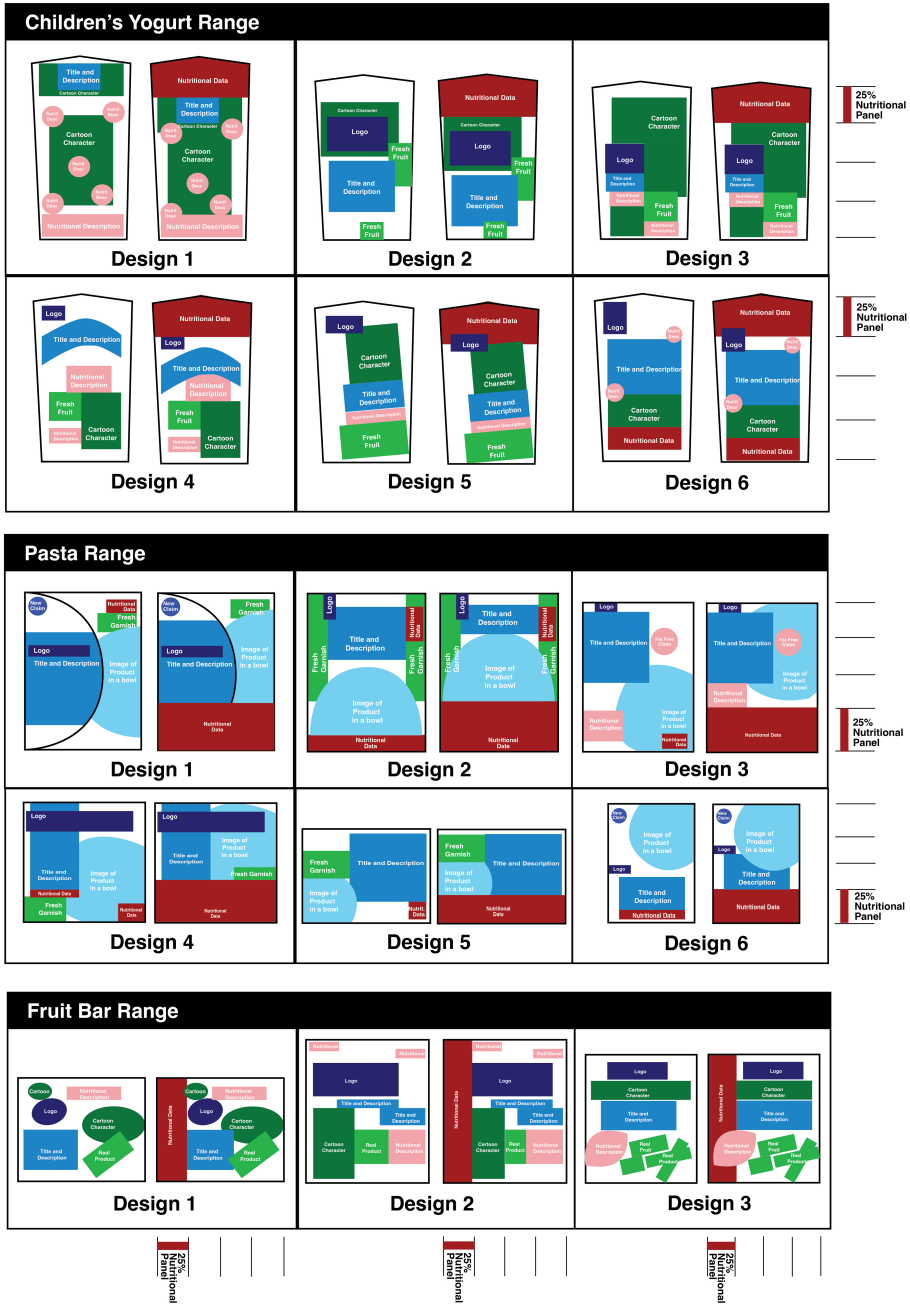
Example of application of a nutrition panel comprising 25% of the pack across different food products within a category using three food categories as exemplars.

Due to the consistencies in design approaches within a food category, it is not difficult to see how this approach could be applied across the range of each food category (Figure 4). The children’s yogurt range shows six designs where the left-hand image highlights the existing design elements, and the right-hand image highlights an increased focus on the essential nutritional information for consumer to make an informed decision. In each instance, the front-of-pack design was not altered with the revised design and instead the existing front-of-pack elements have been moved lower in the package, compacted together in some instances, allowing for an increased visual emphasis, equivalent to one quarter of the package, dedicated to nutrition information. This elevates the nutrition data for the consumer to first in the hierarchy of elements, due to the size and consistent location of the panel of information. Reliably, across all six children’s yogurt range designs, the nutrition panel becomes the first focal point for the consumer, followed by the either the cartoon characters or the title and description.

The ready-made meals achieve the same outcome when the nutrition panel is elevated in the hierarchy to one quarter of the front-of-pack design. The six designs demonstrated below offer, on the left, the original hierarchical arrangement of elements and on the right the revised design, with a focus on allocating 25 % of the package to nutrition information consistently located at the bottom of the front-of-pack design. This is followed by three designs from the fruit bar range where the nutrition information is placed on the left-hand side of the front-of-pack design. With increase of the nutrition element in design hierarchy, the consumer’s ability to identify and read nutrition information should be increased.

## Discussion

In this article we have outlined the significant role that design has in consumer responses to food packaging. Creating a hierarchy of elements visually on a package that prioritizes objective, regulated nutrition information would increase awareness of this content and assist consumers with the visual reading of the information. This would result in stronger consumer awareness and the ability for consumers to make informed decisions. It could lead to new ways of enhancing the impact of regulated nutrition information without compromising the need for visual design to entice consumers. It is important that research demonstrates that designs not only impact consumer attention to nutrition information, but also choice (69). However, the role of hierarchy in design impacting the reading of nutrition information, and influencing decisions and decision-making processes, has not been explored. It is unknown where nutrition information is placed in visual communication hierarchies currently, and whether it is given dominance and emphasis on different types of healthy or unhealthy foods.

There is a need to understand how nutrition information, as an element in a package design, competes with other elements on the package including the title, description of the product and any imagery on current food packages, and how this affects consumer decision making. If nutrition information is given a higher priority compared with other information seen on a front-of-pack (e.g., title, description, images), the hierarchy of elements created visually on a package would increase awareness of the nutrition content and assist consumers with the visual reading of this information. This may disrupt automatic decision-making processes (System 1) and promote the use of rational decision making (System 2) (58). Alternatively, if nutrition information is presented in easy, intuitive ways, then System 1 decision processes could be an effective way to promote healthier choices. This could therefore assist consumers in making informed, accurate decisions based on trustworthy nutrition information. Increasing the impact of nutrition information using a design hierarchy could lead to new ways of enhancing the impact of regulated nutrition information without compromising the need for visual design to entice consumers.

This is not an insurmountable task. As demonstrated above, generalizations can be made in a review of food product categories, one of which is similar packaging and commonly known visual communication strategies are used across a category of food. Although packages look distinctly different across food categories, there are many elements used in a packaging design such as the choice of font, color, and shape of the package that lead to consistency in the design approach within the same food category. This makes the comparative evaluation of food packaging possible. By considering packaging design we can develop novel ways of capturing and directing attention to nutrition information on food packages and thus new approaches for influencing food choices.

Design is not currently considered in a comprehensive way in food/nutrition regulatory frameworks. In Australia, there are currently only regulations that mandate the size and position of some words and elements on a food package, as well as the requirement for a proscribed nutrition information panel (70). However, if evidence accumulates demonstrating how design affects consumption and can be used to promote consumption of healthier foods, this could form the basis of regulatory change. An advantage of this approach is that it is easier to enforce packaging regulation than other domains of food advertising (e.g., digital marketing) (71). Manufacturers who take advantage of this approach could develop a new marketing strategy that may differentiate their product in a saturated market. Companies that are moving toward broader benefits including healthy eating options can maximize their communication strategy through front of pack design. This may generate new ways of addressing the intractable problems of high consumption rates of unhealthy, ultra-processed packaged foods, as part of a wider set of strategies.

## Conclusion

Packaging visual communication design is a neglected health promotion tool. We argue that considering the hierarchy of elements on food packaging designs is likely to enhance the importance of nutritional information by increasing its profile so it may assist consumers in making decisions. Visual communication design hierarchies can therefore help address the fundamental challenge associated with nutrition labelling at present – that many decisions are made without using on-pack nutrition information. Packaging designs that prioritize trustworthy nutrition elements therefore have potential to influence healthy food decisions and improve diet quality and health outcomes for the population.

## Author contributions

MK: Conceptualization, Project administration, Writing – original draft, Writing – review & editing. JM: Writing – review & editing. CC: Writing – review & editing. JW: Writing – review & editing. CR: Conceptualization, Writing – original draft, Writing – review & editing.
